# Towards optimizing peptide-based inhibitors of protein–protein interactions: predictive saturation variation scanning (PreSaVS)†

**DOI:** 10.1039/d1cb00137j

**Published:** 2021-08-16

**Authors:** Kristina Hetherington, Som Dutt, Amaurys A. Ibarra, Emma E. Cawood, Fruzsina Hobor, Derek N. Woolfson, Thomas A. Edwards, Adam Nelson, Richard B. Sessions, Andrew J. Wilson

**Affiliations:** Astbury Centre for Structural Molecular Biology, University of Leeds Woodhouse Lane Leeds LS2 9JT UK a.j.wilson@leeds.ac.uk; School of Chemistry, University of Leeds Woodhouse Lane Leeds LS2 9JT UK; School of Biochemistry, University of Bristol, Medical Sciences Building, University Walk Bristol BS8 1TD UK r.sessions@bristol.ac.uk; School of Molecular and Cellular Biology, University of Leeds Woodhouse Lane Leeds LS2 9JT UK; School of Chemistry, University of Bristol, Cantock's Close Bristol BS8 1TS UK; BrisSynBio, University of Bristol, Life Sciences Building Tyndall Avenue Bristol BS8 1TQ UK

## Abstract

A simple-to-implement and experimentally validated computational workflow for sequence modification of peptide inhibitors of protein–protein interactions (PPIs) is described.

## Abstract 2

An experimentally validated approach for *in silico* modification of peptide based protein–protein interaction inhibitors is described.

## Graphical abstract

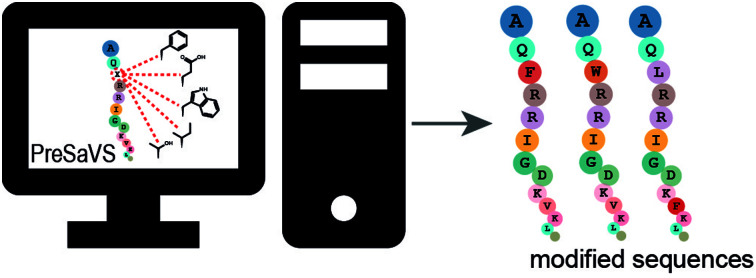

Understanding and modulating PPIs is important both for delineating molecular mechanisms of healthy cells and disease states, and for directing drug discovery.^1–3^ However, PPIs are challenging targets for molecular design.^4^ A significant proportion of PPIs rely on short peptide motifs (SPMs)^5^ for affinity. SPMs are often found in intrinsically disordered regions of proteins^6^ and undergo disorder-to-order transitions to adopt defined structures, *e.g.* α helices^7^ and β-strands,^8^ on interaction with a target domain. The sequences of SPMs serve as powerful templates for inhibitor design, and have motivated efforts to develop peptide and peptidomimetic ligands,^9,10^ including stapled^11^ and macrocyclic peptides,^12^ as potential therapeutics. Strategies for peptide-based ligand development should explore sequence space to maximise binding affinity whilst maintaining good pharmacokinetic properties, such as solubility, cell permeability, and resistance to proteolysis. Concerning affinity, truncation and the identification of hot residues (*i.e.* side chains that contribute significantly to the affinity of a PPI)^13,14^ through alanine scanning^15^ represent practical approaches for obtaining key information on the minimal determinants of binding. Enhancing affinity and/or selectivity through sequence variation is also desirable as exemplified by alternative systematic experimental sequence variation strategies, *e.g.* hydrophile scanning.^16^ However, generally, sequence space is too great too explore using synthetic chemistry. Biological selection methods are powerful and do allow more space to be searched,^17–19^ however, these can be experimentally demanding and expensive. For all these reasons, development and improvement of computational approaches to examine and design PPIs are important.

Computational alanine scanning (CAS) is an important tool within this armoury to speed up and direct experiments.^20,21^ Recent studies show the power of computational design;^22,23^*e.g.*, the use of Rosetta to identify helix bundles that selectively modulate BCL-2 family interactions.^23^ Affinity mapped SORTCERY uses biological selection and deep sequencing to obtain binding information and then develop computational models of sequence-binding relationships to inform peptide design.^24^ Statistical data on tertiary structural motifs (TERMs) in the RCSB Protein Data Bank (PDB) and the resulting TERM energies (dTERMen) have been used in design, predicting peptide binding energies as accurately as structure-based tools, leading to high-affinity peptide binders.^25^ However, these methods rely on large data sets and/or multiple experimental designs being pursued. Rosetta Backrub identifies tolerated sequences using flexible backbone protein design. It uses a simulated annealing and genetic algorithm optimization method to create a single estimate for the tolerated sequence space.^26–28^ Finally, AlphaSpace can be used to identify unoccupied spaces in PPIs, which can be filled by natural or unnatural side chains in designs targeting interfaces.^29^

Here, we describe a simple-to-implement and experimentally validated computational workflow for sequence modification of peptide-based PPI inhibitors. We call this *in silico* Predictive Saturation Variation Scanning (PreSaVS). Rather than knocking-out affinity (as in CAS), modifying a sequence whilst retaining or even improving its potency is challenging: it is unclear if further optimization of hot residues, or making new interactions using non-hot residues^30^ is the best approach and to what extent affinity can be increased. PreSaVS computationally substitutes each residue in a peptide sequence of interest to 16 of the proteinogenic amino acids (*i.e.*, standard residues except Ala, Gly, Pro and Cys) and calculates the difference in binding free energy (ΔΔ*G*) relative to the native sequence (Fig. 1a). To do this, we adapted BUDE alanine scanning^20,31^ to allow variation to different amino acids (see ESI†). The soft nature of the BUDE forcefield avoids penalising small geometric overlaps in the modelled structures and this is key to the speed of these methods, unlike the Rosetta forcefield, where extensive sampling is required.^20^ To establish the PreSaVS workflow, it was applied to both α helix- and β strand-mediated PPIs (Fig. 1b–i). A modified *m*NOXA-B/*h*MCL-1^32,33^ interaction (referred to as NOXA_75–93_/MCL-1) was selected as the model α-helix-mediated PPI (Fig. 1b). NOXA and MCL-1 are proteins of the B-cell Lymphoma 2 (BCL-2) family of apoptosis regulators,^34^ and have been the focus of oncology drug-discovery efforts.^35^ The interaction between the SIM peptide found in the M-IR2 region of RanBP2, and *h*SUMO−1 (referred to as SIM_2705–2717_/SUMO)^36^ was chosen as the model β strand-mediated PPI (Fig. 1f). Small ubiquitin-like modifiers (SUMO) regulate many cellular processes through their interaction with SUMO-interacting motifs (SIMs) within other proteins, and are the subject of ongoing investigation.^37^

**Fig. 1:**
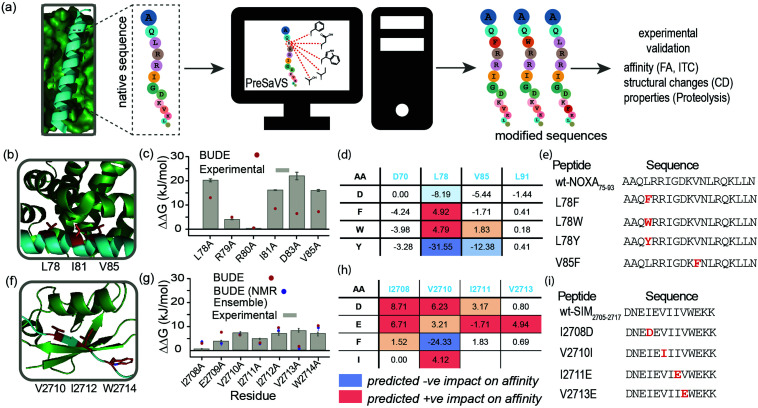
PreSaVS applied to NOXA_75–93_/MCL-1 and SIM_2705–2717_/SUMO. (a) Schematic depicting workflow; (b) lowest energy NMR-derived structure (PDB ID: 2JM6) of *m*NOXA_68–93_ (cyan)/*m*MCL-1 (green, hot residues in red); (c) BudeAlaScan and experimental data for NOXA_75–93_/MCL-1; (d) key PreSaVS results for NOXA_75–93_; (e) NOXA_75–93_ sequences selected for experimental analyses; (f) lowest energy NMR derived structure (PDB ID: 2LAS) of SIM_2705–2717_ (cyan)/SUMO (green, hot residues in red); (g) BudeAlaScan and experimental data for SIM_2705–2717_/SUMO; (h) key PreSaVS results for SIM_2705–2717_; (i) SIM_2705–2717_ sequences selected for experimental analyses.

By analogy to hot residues (ΔΔ*G* ≥ −4.5 kJ mol^−1^), we set ΔΔ*G* ≥ 4.5 kJ mol^−1^ ^13,14^ as a threshold for experimental analyses. The two PPIs were subjected to PreSaVS and the outcomes visually inspected (Fig. 1d and h; full data in ESI,† Tables S1 and S2). Based on prediction of a favourable increase in affinity, we selected NOXA_75–93_ peptides with substitutions at L78 and V85 (both hot residues, Fig. 1b and e) for experimental analyses, specifically focussing on Trp and Phe variants. For L78, we also selected the Tyr variant as a negative control. Predictions for SIM_2705–2717_ identified hot and non-hot residues, focussing predominantly on substitution of hydrophobic for charged amino acids (Fig. 1f–i): I2708D, V2710I (hot residue), I2711E and V2713E (hot residue) variations were selected for experimental validation.

Peptide variants were prepared as N-terminal acetamides and C-terminal amides (see ESI†). These were tested in fluorescence anisotropy (FA) competition assays using a fluorescein-labelled BIM_75–85_ peptide (FITC-Ahx-BIM_75–85_, *K*_d_ = 204 ± 16 nM) or SIM_2705–2717_ (FITC-peg-SIM_2705–2712_, *K*_d_ = 1.5 ± 0.2 μM) peptides described previously (see ESI† for sequences and direct titration data, Fig. S1).^20^ Competition FA revealed that NOXA_75–93_L78F and NOXA_75–93_L78W variations were tolerated, whilst the NOXA_75–93_V85F variant displayed weaker inhibitory activity (Fig. 2a). As predicted by PreSaVS, the NOXA_75–93_L78Y variant was a poor inhibitor providing confidence in the predictions. The NOXA_75–93_ peptide binds MCL-1 selectively over other BCL-2 family members,^38^ and peptide variants retained this selectivity in a FA competition assay against a further BCL-2 family member protein, BCL-x_L_ (Fig. S2, ESI†). Isothermal calorimetry (ITC) experiments showed comparable *K*_d_ values for the variant peptides relative to NOXA_75–93_, confirming these results; variant peptides exhibited a lower entropy and compensatory decrease in enthalpy of binding compared to NOXA_75–93_ (Fig. 2b, Table 1 and Fig. S3, ESI†). Lastly, structural effects of these variations were investigated using circular dichroism (CD) spectroscopy (Fig. S4, ESI†). The peptides exhibited CD spectra consistent with predominance of random coil in buffer (peptides exhibited increased helicity in 30% trifluoroethanol, Fig S5, ESI†).^45^ The sequence-driven variation in helicity is greater than the variation in affinity (see Fig. S6, ESI†) although both are small supporting the hypothesis that side chain interactions dominate the observed effects of variation as opposed to a significant change in conformational preference.

**Fig. 2:**
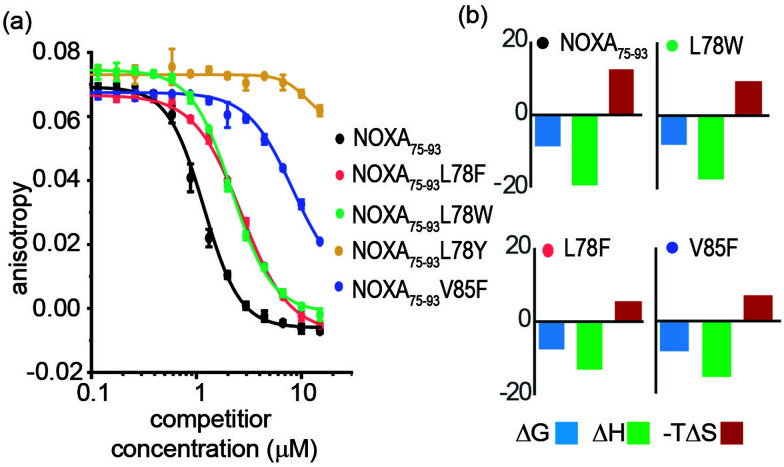
FA competition and ITC data for NOXA_75–93_ and variant sequences. (a) Competition FA (50 mM Tris, 150 mM NaCl, pH 7.5, using 25 nM tracer and 150 nM MCL-1). (b) ITC data (50 mM Tris, 150 mM NaCl, pH 7.5, 150 μM peptide).

**Table 1:** MCL-1 binding parameters and helicities for NOXA_75–93_ peptides^a^

	NOXA_75–93_	L78F	L78W	L78Y	V85F
*K*_d_ (μM)	0.4 ± 0.1	1.7 ± 0.1	2.4 ± 0.8	—	0.8 ± 0.1
IC_50_ (μM)	1.2 ± 0.1	2.7 ± 0.1	2.2 ± 0.1	>100	>20
Δ*G*^b^	−8.71	−7.88	−7.66	—	−8.35
Δ*H*^b^	−19.2 ± 1.5	−17.3 ± 3.2	−13.1 ± 3.9	—	−15.3 ± 3.4
−*T*Δ*S*^b^	12.2	9.44	5.48	—	6.90
% helicity^c^	15	8	12	—	9

a Determined using conditions as noted in Fig. 2.

b (kJ mol^−1^).

c In buffer.

For SIM_2705–2717_ peptide variants (Fig. 3a and Table 2), similar inhibitory potency was observed for SIM_2705–2717_I2708D (IC_50_ = 14.8 ± 0.7 μM) in comparison to SIM_2705–2717_ (IC_50_ = 21.9 ± 0.3 μM). In the bound state, the hydrophobic I2708 SIM side chain lies proximal to K45 and K46 of SUMO; swapping this position for Asp may introduce hydrogen-bonding interactions (Fig. 3b). Less surprising was the more conservative SIM_2705–2717_V2710I variant peptide, which maintained SUMO binding with an IC_50_ = 16.2 ± 1.6 μM. (Fig. 3a). Finally, the SIM_2705–2717_I2711E and SIM_2705–2717_V2713E variants lost inhibitory potency. In the NMR ensemble, I2711 rests against the surface of the SUMO protein between the hydrophobic Y21 and the charged K37 residues of (Fig. 3b), implying any benefit from introduction of a salt bridge may be countered by repulsion from the electron-rich aromatic ring. Lastly, the V2713 side chain is solvent exposed (Fig. 3b), so the weaker inhibitory potency of the V2713E variant cannot be reconciled by considering potential interactions it may make with the SUMO interface. Alanine scanning, however, previously showed loss in binding for V2713A.^20^ Valine favours β structure,^39^ and we attribute the loss of affinity observed for V2713E to a critical structure imposing role for V2713 in adopting a compliant SUMO-binding conformation. CD spectra on SIM peptides were consistent with a random coil conformation as expected (not shown).

**Fig. 3:**
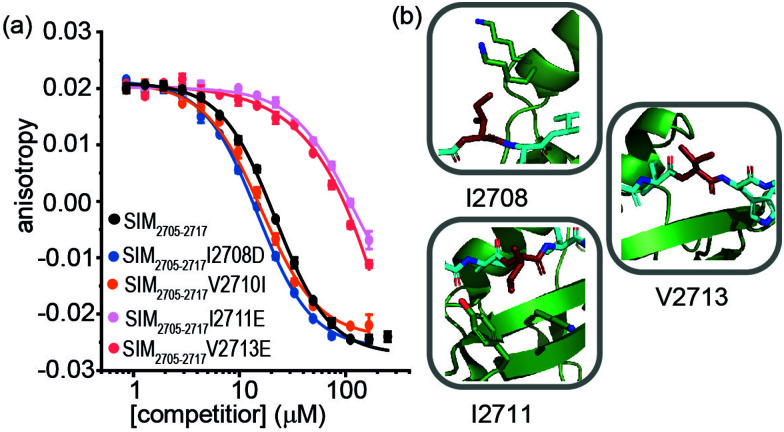
FA competition data and structural analysis for SIM_2705–2717_ and variant sequences; (a) competition FA (50 mM Tris, 150 mM NaCl, 25 nM tracer, 100 nM SUMO; (b) SIM_2705–2717_/SUMO (PDB ID: 2LAS) variant residues highlighted in red showing I2708 proximity to SUMO K45 and K46 residues, I2711 proximity to SUMO residues Y21 and K37 and V2713 pointing towards solvent.

**Table 2:** SUMO binding parameters for SIM_2705–2717_ variant peptides^a^

	SIM_2705–2717_	I2708D	V2710I	I2711E	V2713E
IC_50_ (μM)	21.9 ± 0.3	14.8 ± 0.7	16.1 ± 1.6	>200	>200

a Determined using conditions as noted in Fig. 3.

An advantage of PreSaVS lies in the ability to identify tolerated variants – in terms of target binding affinity – with more desirable physicochemical properties (Table S3 for calculated physicochemical properties, ESI†). Notable changes were observed in proteolysis studies. Both NOXA_75–93_ variants tested (L78F and L78Y) exhibited ≈10-fold increased rate of cleavage by α-chymotrypsin relative to NOXA_75–93_ (Fig. S7a, ESI†). In contrast, SIM variants showed improved Proteinase K stability over SIM_2705–2717_ (Fig S7b, ESI†), with the greatest protection observed for the V2713E variant (≈9-fold decreased proteolysis rate). For NOXA_75–93_, while the peptide bond between residues L78 (P1) and R79 (P1′) is a substrate for α-chymotrypsin, cleavage susceptibility for peptides containing Leu, Phe, or Tyr at the P1 position is similar.^40^ Although small, the increased helical propensity of NOXA_75–93_ (Fig. S4 and S5, ESI†) may contribute to its higher protease resistance compared to the variant sequences. The broad-range specificity of Proteinase K,^40^ renders most peptide bonds in the SIM_2705–2717_ sequence targets for this protease. The relative Proteinase K resistance of these peptides (SIM_2705–2717_ ∼ I2708D ∼ V2710I < I2711E < V2713E) is similar to their relative affinity for SUMO. This may arise from subtle changes in protease recognition specificity, although given proteases recognise substrates in an extended, β-like conformation as is the case for the SIM_2705–2717_/SUMO interaction, secondary structure propensity may also play a role.

## Conclusions

We have developed *in silico* Predictive Saturation Variation Scanning (PreSaVS) and tested it on α helix- and β strand-mediated PPIs. Variants of NOXA_75–93_ and SIM_2705–2717_ peptides generated by PreSaVS retained inhibitory potency/affinity for MCL-1 and SUMO, respectively. Tolerated modifications could be made at hot and non-hot residues. Further experiments revealed changes in proteolysis rates of the peptides as a consequence of the sequence variation. Whereas suppressed proteolysis is often desired, accelerated proteolysis can be advantageous for fast-acting peptides.^41^ This validates PreSaVS as a fast predictive tool for sequence variation and further extends the capabilities of the Bristol University Docking Engine (BUDE).^42^ We expect the approach to be useful for other topologies *e.g.* loops, and, whilst NOXA and SIM are intrinsically disordered their PPIs are well defined; thus utility of the approach for “fuzzy” interactions remains to be explored. Ongoing studies are focussed on exploring the scope to identify affinity enhancing and/or selectivity modifying variations across a broader array of PPI targets.^43^

## Author contributions

D. N. W., T. A. E., A. N., R. B. S., and A. J. W., conceived and designed the research program, K. H. and S. D. prepared and tested peptides, A. A. I. developed the computational workflow, E. E. C carried out proteolysis, F. H. produced protein and carried out ITC. The manuscript was written by K. H. and A. J. W. with contributions from all authors.

## Conflicts of interest

There are no conflicts to declare.

## Supplementary Material

CB-002-D1CB00137J-s001
