# Supplementary material for: Towards optimizing peptide-based inhibitors of protein–protein interactions: predictive saturation variation scanning (PreSaVS)
Source: RSC Chem Biol. 2021 Aug 16;2(5):1474–8. doi: 10.1039/d1cb00137j (PMC8495968; doi:10.1039/d1cb00137j)
Supplement: CB-002-D1CB00137J-s001 [file CB-002-D1CB00137J-s001.pdf]

Electronic Supplementary Material (ESI) for RSC Chemical Biology.
This journal is © The Royal Society of Chemistry 2021

Towards Optimizing Peptide Based Inhibitors of Protein-Protein Interactions: Predictive Saturation Variation Scanning (PreSaVS)

Kristina Hetherington,[a,b]† Som Dutt,[a,b]† Amaurys A. Ibarra,[c]† Emma E. Cawood,[a,b] Fruzsina Hobor,[a,d] Derek N. Woolfson,[c,e,f] Thomas A. Edwards,[a,d] Adam Nelson,[a,b] Richard B. Sessions,*[c,f] Andrew J. Wilson* [a,b]

[a]Astbury Centre for Structural Molecular Biology, University of Leeds, Woodhouse Lane, Leeds LS2 9JT, UK
[b] School of Chemistry, University of Leeds, Woodhouse Lane, Leeds LS2 9JT, UK
[c]School of Biochemistry, University of Bristol, Medical Sciences Building, University Walk, Bristol BS8 1TD, UK
[d]School of Molecular and Cellular Biology, University of Leeds, Woodhouse Lane, Leeds LS2 9JT, UK
[e]School of Chemistry, University of Bristol, Cantock's Close, Bristol BS8 1TS, UK
[f]BrisSynBio, University of Bristol, Life Sciences Building, Tyndall Avenue, Bristol BS8 1TQ, UK

Electronic Supporting Information

Contents

Computational Methods 7
Solid Phase Peptide Synthesis 7
General Remarks: 7
Cycles for Automated Peptide Synthesis: 7
Deprotection and Coupling 7
Deprotection Microwave Methods: 8
Coupling Methods: 8
Tracer peptides 8
Kaiser Test 8
N-terminal acetylation 9
Cleavage and deprotection of Rink amide MBHA resin 9
Peptide Purification 9
Peptide Characterization Data 9
Protein Expression and Purification 10
Fluorescence Anisotropy 10
Direct binding assays: 11
Competition binding assays: 11
Isothermal Calorimetry 11
Circular Dichroism 11
Solubility Analyses 11
Proteolysis 11
Peptide Analytical Data: 13
NOXA-B peptides 13
SIM peptides 14
References 16

Supplementary Figures

AA	D	E	F	H	I	K	L	M	N	Q	R	S	T	V	W	Y
P68	2.97	3.61	1.87	0.88	1.96	-0.20	0.83	0.80	1.26	0.53	0.22	-0.07	1.10	1.31	0.50	3.56
A69	1.87	6.18	1.10	0.10	0.38	-3.72	0.59	0.17	0.41	0.58	-1.93	0.12	0.23	0.28	0.27	1.40
D70	0.00	0.96	-4.24	-4.13	-2.61	-5.89	-4.89	-5.40	-6.36	-3.52	-8.23	-4.94	-3.22	-3.18	-3.89	-3.28
L71	5.07	7.70	4.57	3.64	0.85	-8.37	0.00	-1.64	-5.43	-1.26	-4.89	-4.13	-0.63	-2.23	9.98	4.62
K72	6.23	5.68	2.60	1.35	2.33	0.00	2.29	2.00	2.35	1.82	1.73	1.64	1.88	1.92	3.72	2.80
D73	0.00	0.81	-4.35	-5.35	-4.02	-8.12	-4.49	-5.59	-6.32	-4.11	-7.77	-5.99	-4.92	-5.15	-4.12	-4.34
E74	-7.77	0.00	-35.62	-25.88	-16.01	-25.51	-14.18	-16.66	-13.98	-14.84	-20.67	-19.00	-21.74	-19.95	-60.60	-55.82
C75	6.35	1.88	-22.50	5.85	4.22	-16.50	-18.20	-8.78	-2.60	3.12	-2.64	3.57	5.74	4.90	1.31	11.52
A76	-0.91	-0.04	-2.40	-1.10	0.41	-0.79	-2.02	0.09	-2.43	0.44	-0.13	-1.21	-0.46	0.52	0.54	-2.30
Q77	-0.01	0.73	0.80	-0.18	1.00	-0.37	0.59	0.23	-1.68	0.00	0.88	-1.48	0.04	0.13	2.25	0.81
L78	-8.19	-3.10	4.92	5.34	-1.45	-3.06	0.00	-4.25	-7.06	-2.97	-6.83	-11.60	-11.92	-7.21	4.79	-31.55
R79	-22.00	-23.57	-20.71	-14.08	-8.68	6.25	-10.76	-12.12	-12.08	-8.42	0.00	-9.35	-12.00	-11.52	-19.10	-25.38
R80	-0.12	0.40	-0.26	-0.87	0.55	0.37	0.47	0.37	0.40	0.41	0.00	-0.44	-0.28	0.53	-0.30	1.33
I81	-0.75	-3.75	-8.85	-5.66	0.00	-10.12	-1.45	-6.70	-2.37	-2.75	-10.45	-6.78	-3.88	-2.87	-33.18	-12.52
G82	-20.20	-2.76	-93.64	-41.30	-35.17	-21.88	-41.18	-27.72	-26.10	-0.48	-25.01	3.89	-3.67	-13.68	-47.50	-30.74
D83	0.00	-4.89	-8.52	-13.06	-9.69	-9.15	-8.75	-9.92	-8.68	-9.32	-13.74	-8.69	-8.46	-10.68	-9.06	-8.71
K84	-1.20	-1.24	-1.35	0.27	-6.92	0.00	-5.64	-4.99	-1.38	-2.00	-1.62	-5.90	-4.21	-4.57	2.50	-0.90
V85	-5.44	-2.07	-1.71	-2.87	0.74	1.84	-2.01	-3.52	-4.05	-1.96	-0.81	-7.29	-4.25	0.00	1.83	-12.83
N86	9.12	8.97	5.79	-0.63	-1.97	-4.13	1.25	2.11	0.00	1.70	-3.25	1.84	2.05	-1.83	2.64	7.54
L87	0.05	-0.36	0.16	0.02	-0.63	-0.02	0.00	0.00	0.05	-0.26	0.32	-0.03	-0.03	-0.62	-0.45	0.15
R88	-12.44	-12.61	-4.45	-2.90	-5.31	2.53	-6.83	-6.24	-6.69	-5.24	0.00	-7.57	-5.70	-5.51	0.43	-3.53
Q89	-8.57	-6.87	-3.78	-7.85	-2.18	3.56	-4.96	-1.51	-4.87	0.00	1.00	-7.84	-6.17	-2.70	-1.11	-9.74
K90	-0.16	-0.03	0.47	-0.06	-0.83	0.00	0.28	-0.09	-0.12	-0.02	-1.09	-0.59	-0.46	0.05	1.35	0.47
L91	-1.44	-2.71	0.41	0.16	0.47	3.58	0.00	-0.09	-0.09	-0.09	0.66	-0.12	0.02	0.18	0.18	0.41
L92	-12.65	-6.90	-1.80	3.38	1.76	13.91	0.00	3.93	1.47	3.68	8.37	1.15	2.56	2.94	-5.85	-3.26
N93	-6.92	-4.80	-1.72	5.16	-1.36	7.39	-0.41	-1.86	0.00	0.49	5.79	-2.23	-0.59	0.13	2.71	-11.18
M94	-1.13	-0.11	1.21	1.86	0.20	2.08	0.65	0.00	-0.19	0.81	0.29	1.29	-0.36	-0.04	1.58	1.55

Table S1. Complete PreSaVS table containing the results for the $NOXA_{75\text{-}93}$ peptide.

AA	D	E	F	H	I	K	L	M	N	Q	R	S	T	V	W	Y
D2705	0.00	-0.11	-0.83	-1.14	-1.04	-0.14	-0.91	-0.91	-1.73	-0.80	-0.71	-1.41	-1.48	-1.66	-0.77	-0.63
N2706	1.18	1.42	1.05	0.88	0.59	1.07	0.70	0.63	0.00	0.85	0.61	0.45	0.53	0.57	0.92	1.12
E2707	0.98	0.00	-0.04	-0.48	-0.14	-0.41	-0.31	-0.29	-0.38	-0.14	-0.77	-0.50	-0.39	-0.24	0.87	-0.04
I2708	3.65	2.87	0.65	0.03	0.00	1.97	0.05	-1.36	1.21	0.98	0.91	-1.08	0.30	-0.12	2.60	1.72
E2709	-0.03	0.00	-1.88	-2.38	-1.42	-1.53	-1.73	-1.92	-1.60	-0.42	-1.49	-2.07	-1.56	-1.63	-1.38	-1.62
V2710	-0.28	1.25	-20.47	-1.18	3.56	-0.84	-1.05	-2.12	1.70	2.17	-4.84	-2.41	-0.57	0.00	-32.56	-32.52
I2711	-0.64	0.85	0.90	-1.42	0.00	-2.59	-0.21	-0.20	-0.35	0.68	-1.92	-1.75	-1.10	-0.36	0.42	1.07
I2712	-6.49	-5.05	-3.93	-5.06	0.00	-4.48	-6.37	-7.20	-2.38	0.46	-5.77	-5.83	-3.16	-1.25	-45.78	-39.68
V2713	0.80	1.38	0.76	-0.37	0.53	-0.92	0.40	0.17	0.10	0.65	-0.78	-0.31	0.16	0.00	0.90	0.75
W2714	-5.19	-4.05	-0.49	-5.50	-2.53	-6.00	-10.73	-5.22	-7.02	-4.27	-4.98	-6.43	-9.82	-4.34	0.00	-0.06
E2715	-0.62	0.00	-0.64	-0.33	-0.65	-0.08	-0.86	-0.85	-0.91	-0.79	-0.57	-1.06	-1.04	-0.83	-0.69	-0.63
K2716	-0.22	-0.23	0.80	-0.27	-0.28	0.00	0.55	-0.17	-0.39	-0.05	-0.03	-0.51	0.04	-0.34	1.77	1.07
K2717	-1.74	-1.63	-1.06	-0.41	-1.20	0.00	-1.06	-1.51	-1.82	-1.22	0.08	-1.93	-1.32	-1.36	0.07	-0.81

Table S2. Complete PreSaVS table containing the results for the $SIM_{2705\text{-}2717}$ peptide

Peptide	Sequence	Theoretical pI	GRAVY	Estimated

				$t_{1/2}$[a]
NOXA$_{75\text{-}93}$	AAQLRRIGDKVNLRQKLLN	11.72	-0.616	4.4 h
NOXA$_{75\text{-}93}$L78F	AAQ**F**RRIGDKVNLRQKLLN	11.72	-0.668	4.4 h
NOXA$_{75\text{-}93}$L78W	AAQ**W**RRIGDKVNLRQKLLN	11.72	-0.863	4.4 h
NOXA$_{75\text{-}93}$L78Y	AAQ**Y**RRIGDKVNLRQKLLN	11.00	-0.884	4.4 h
NOXA$_{75\text{-}93}$V85F	AAQLRRIGDK**F**NLRQKLLN	11.72	-0.689	4.4 h
SIM$_{2705\text{-}2717}$	DNEIEVIIVWEKK	4.41	-0.331	1.1 h
SIM$_{2705\text{-}2717}$I2708D	DNE**D**EVIIVWEKK	4.18	-0.946	1.1 h
SIM$_{2705\text{-}2717}$V2710I	DNEIE**I**IIVWEKK	4,41	-0.308	1.1 h
SIM$_{2705\text{-}2717}$I2711E	DNEIEV**E**IVWEKK	4.25	-0.946	1.1 h
SIM$_{2705\text{-}2717}$V2713E	DNEIEVII**E**WEKK	4.25	-0.923	1.1 h

Table S3. Properties Calculated using the EXPASY ProtParam Tool[1] (GRAVY = Grand average of hydropathicity)[2] [a] mammalian reticulocytes, in vitro. All peptides were highly soluble: unsurprisingly, no variation in solubility was observed for NOXA peptides, although for SIM variants higher concentrations could be obtained (e.g. SIM$_{2705\text{-}2717}$ I2708D > 8 mM v SIM$_{2705\text{-}2717}$ maximum solubility ~1 mM consistent with the diminished hydrophobic character).

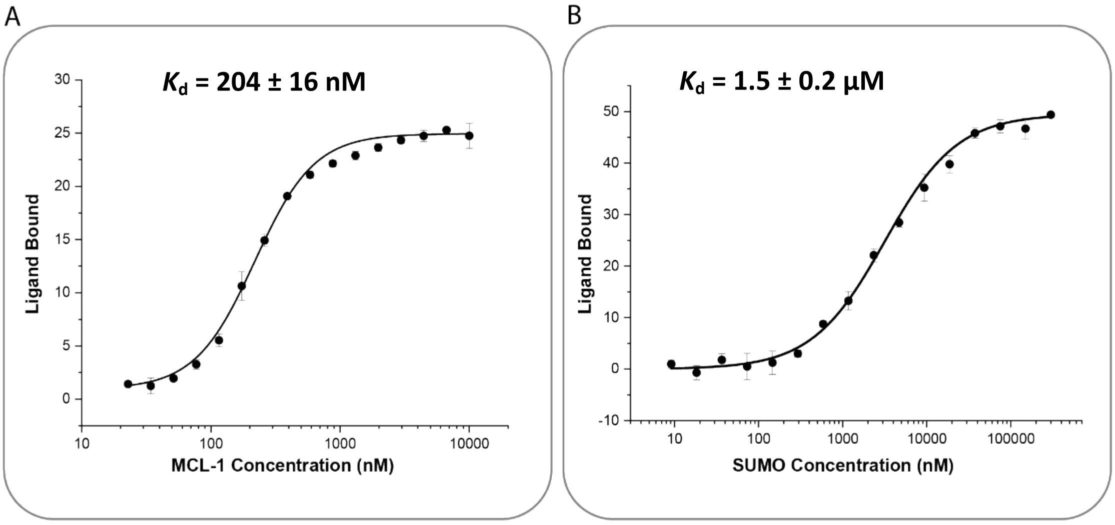

FITC-Ahx-BIM$_{75\text{-}85}$ = FAM-Ahx-EDIIRNIARHLAQVGDSMDRSIW

FITC-PEG-SIM$_{2705\text{-}2712}$ = FAM-peg-DNEIEVIIVWEKK

peg = Amino-3,6 dioxaoctanoic acid

Figure S1. FA direct titration curves. A. Direct titration assay for NOXA$_{75\text{-}93}$/MCL-1 in 50 mM Tris, 150 mM NaCl, pH 7.5 using 25 nM tracer concentration B. Direct titration assay for SIM$_{2705\text{-}2717}$/SUMO in 50 mM Tris, 150 mM NaCl, pH 7.5 using 50 nM tracer concentration.

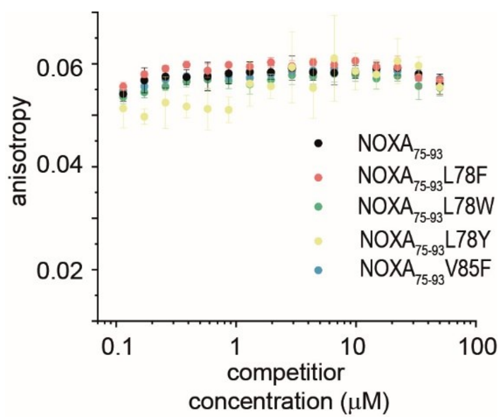

Figure S2. FA Competition assays for the $NOXA_{75\text{-}93}$ variant peptides against the BAK/BCL-x_L interaction.

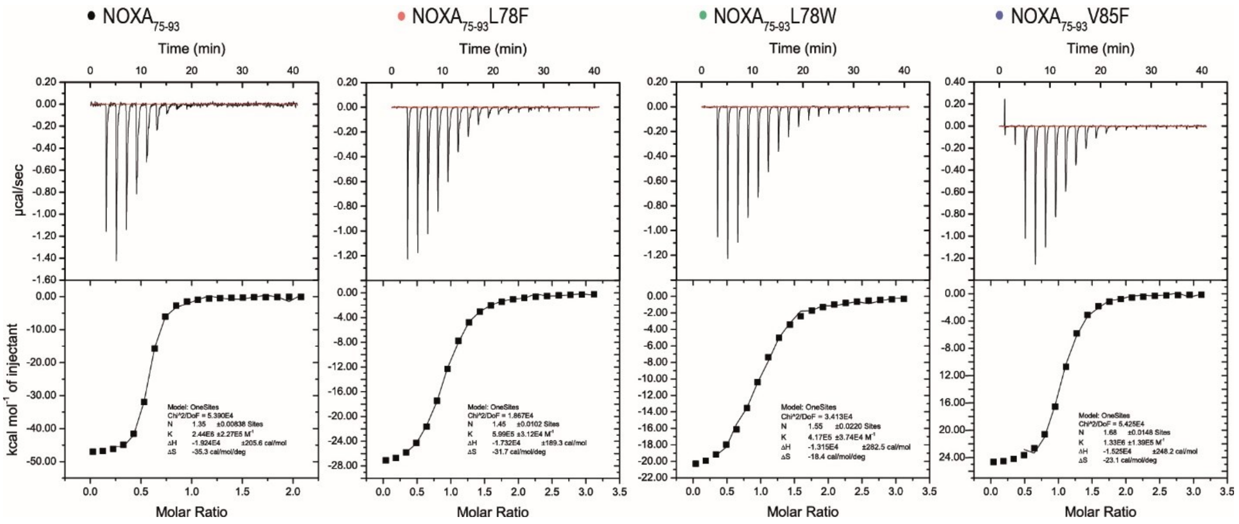

Figure S3. ITC thermograms for binding of $NOXA_{75\text{-}93}$ peptide towards MCL-1

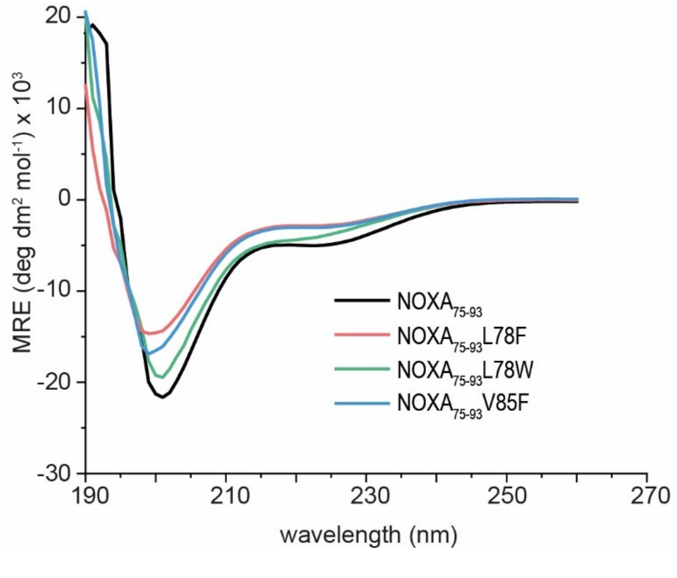

Figure S4. CD data for $NOXA_{75\text{-}93}$ variants: CD spectra measured in 20 mM potassium phosphate, pH 7.5 at 150 μM peptide concentration)

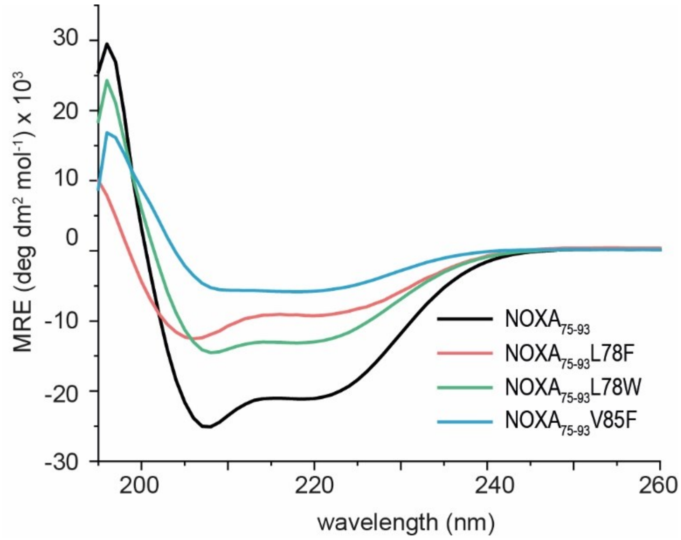

Figure S5. CD data for $NOXA_{75\text{-}93}$ peptide variants: CD spectra measured in 30% TFE in 20 mM potassium phosphate, pH 7.5 at 150 μM peptide concentration.

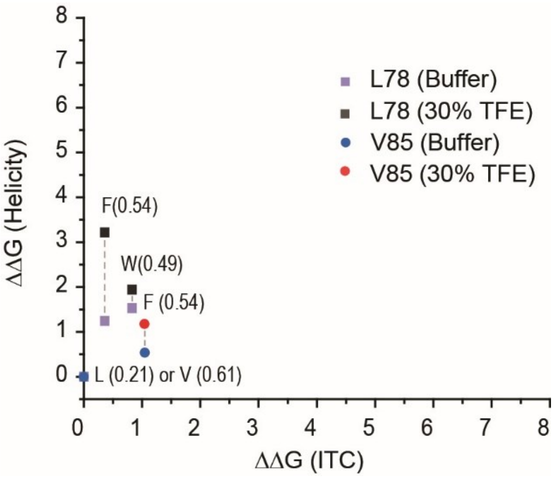

Figure S6. Analysis of the relationship between helicities determined from CD data and MCL-1 binding affinity for $NOXA_{75\text{-}93}$ variants (values in kJ/mol). Squares are L78 variants, circles denote V85 variants, numbers in brackets denote fractional helical propensities of amino acid at the stated position).[3] The data suggest that small decreases in helicity areobserved for all variant peptides regardless of the helical propensity of the variant amino acid, reinforcing the conclusion that the amino acid is tolerated in terms of MCL-1 recognition.

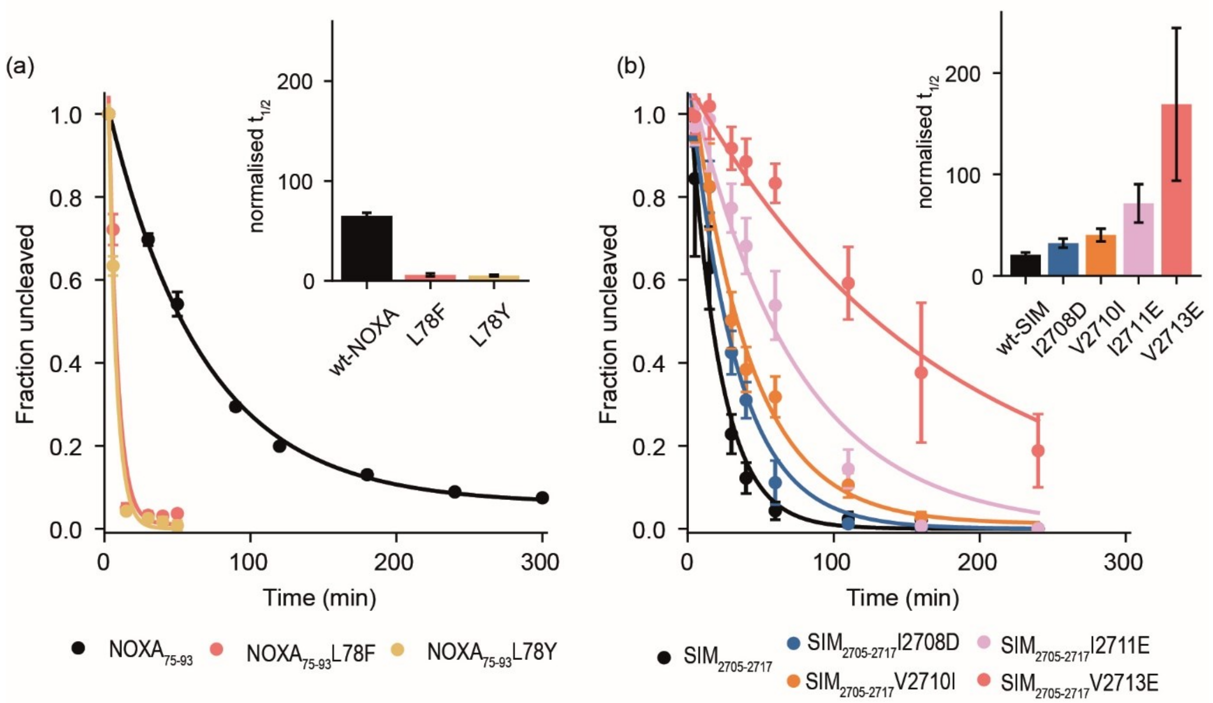

Figure S7. Proteolysis data for (a) $NOXA_{75-93}$ (45 μM peptide in the presence of 0.25 μg/ml α-chymotrypsin at 25 °C) and (b) $SIM_{2705-2717}$ (40 μM peptide in the presence of 0.75 μg/mL Proteinase K at 25 °C). Data points show the mean ± standard deviation over triplicate measurements. Insets show $t_{1/2}$ values.

Computational Methods

Input for PreSaVS is simply a PDB file of the complex in question. We used the first models of the NMR structures of the NOXA-B/MCL-1 complex (2JM6) and the SIM/SUMO complex (2LAS). A program, saturation_mutagenesis.py, was written in Python3 to perform PreSaVS, leveraging functions and methods from BudeAlaScan.[4] The data presented here were generated with the two commands:

saturation_mutagenesis.py full -v -p 2jm6.pdb -l A > 2jm6_full.log

saturation_mutagenesis.py full -v -p 2las.pdb -l B > 2las_full.log

By default, the method requires a PDB file with two protein chains and identifies the interfacial residues between them. Each interfacial residue position is replaced with one of 16 proteinogenic amino acids (i.e., standard residues except Ala, Gly, Pro and Cys, noting Ala is covered under BudeAlaScan/BAlaS)[4, 5] and scored with the BUDE forcefield with the rotamer correction previously described.[4] Each substitution gives an interaction energy and subtracting this from the interaction energy of the native residue gives a value in kJ/mol, such that a residue stabilising the interface with respect to the native residue will give a positive value

The software is available on demand *via* github see:

https://github.com/richardbsessions/BUDE_SM

Solid Phase Peptide Synthesis

General Remarks:

All amino acids and resins were purchased from either Novabiochem (Merck) or Sigma-Aldrich. All amino acids were *N*-Fmoc protected and side chains protected with Boc (Lys); O^{t}Bu (Asp, Ser, Thr); Trt (Asn, Gln); Pbf (Arg). Synthesis of all peptides was performed using a microwave assisted automated peptide synthesiser (CEM, Liberty or Liberty Blue). Coupling of 6-aminohexanoic acid, γ-aminobutyric acid and N-terminal labelling were performed manually. DMF used in peptide synthesis was of ACS grade and from Sigma Aldrich. Peptides were synthesised on an 0.1 mmol scale and split before acetylation and fluorescent ligation. Lyophilisation was performed using a BenchTop Pro with Omnitronics^{TM} from VirTis SP Scientific. Preparative HPLC was performed on an Agilent Technologies 1260 infinity controller in conjunction with a diode array detector. Analytical HPLC was performed on an Agilent Technologies 1260 infinity controller in conjunction with a diode array detector. Mass spectrometry data were obtained on a Bruker Daltonics micrOTOF using electrospray ionisation (ES)MS.

Cycles for Automated Peptide Synthesis:

Peptides that were prepared on a microwave assisted Liberty CEM peptide synthesiser followed this cycle:

Resin Loading – Clean reaction vessel; wash with DMF, wash with CH_2Cl_2; transfer resin to reaction vessel; wash with DMF, wash with CH_2Cl_2; vessel draining.

Deprotection and Coupling

Clean resin dip tube, wash with DMF (15 mL) add 20% piperidine in DMF (6 mL), microwave method (30 sec), wash with DMF (15 mL), clean resin dip tube, wash with DMF (15 mL), add amino acid (2.5 mL), add coupling reagent (1 mL), add activator base (0.5 mL), microwave method (5 min), wash with DMF (15 mL), drain.

For methods that ***did not*** use microwave assistance, the reaction cycle was the same, expect the microwave method for deprotection and coupling was replaced by agitation of the resin at rt for 10 min and 90 min respectively.

After the final residue, the resin was ejected from the reaction vessel and linker coupling, capping, cleavage and deprotection was performed manually.

Peptides that were prepared on the microwave assisted Liberty Blue CEM peptide synthesiser followed this cycle:

After the final residue, the resin was ejected from the reaction vessel and linker coupling, capping, cleavage and deprotection was performed manually using methods A to F

For the microwave methods used, the temperature and total time is shown below:

Deprotection Microwave Methods:

Method	Ramp Time	Total Time	Max Temp
Standard	20-30 sec	1:05	90 °C
75 °C deprotection*	30 sec	0:30	~50 °C
	30-75 sec	3:00	75 °C
Conventional*	N/A	5:00	rt
	N/A	10:00	rt

Coupling Methods:

Method	Ramp Time	Total Time	Max Temp
Standard	20-30 sec	1:05	90 °C
50 °C MW	N/A	2:00	rt
	30-75 sec	4:00	50 °C
Arg* coupling	N/A	25:00	rt
	30-75 sec	2:00	75 °C
75 °C coupling	30 sec	0:30	~50 °C
	30-75 sec	5:00	75 °C

*Methods for double deprotection/coupling

Tracer peptides

Tracer peptides with the sequences shown below bearing a fluorescein linked to the *N*-terminus *via* an aminohexanoic acid linker were described previously.[4]

FITC-Ahx-BIM_{75-85} = `FAM-Ahx-EDIIRNIARHLAQVGDSMDRSIW`

FITC-PEG-$SIM_{2705-2712}$ = `FAM-peg-DNEIEVIIVWEKK`

peg = Amino-3,6 dioxaoctanoic acid

Kaiser Test

The Kaiser Test[6] was used for the determination of the successful coupling or deprotection for any residue coupled manually. A small number of resin beads were rinsed with CH_2Cl_2 and placed in a vial, followed by the addition of two drops of each of the three solutions below:

1) Ninhydrin (5% w/v) in ethanol

2) Phenol (80% w/v) in ethanol

3) 1 mM KCN (aq.) in pyridine (2% v/v)

The solution was then heated to ca. 100°C for five minutes. A successful coupling gave no change in the colour of the beads, whereas bright blue beads demonstrated a successful deprotection.

N-terminal acetylation

Acetic anhydride (10 equiv.) and DIPEA (10 equiv.) were dissolved in DMF (1 mL) and the solution was transferred to the resin. After 2 h, the resin was drained, washed with DMF (3 × 2 mL × 2 min) and successful capping determined by a negative Kaiser test.

Cleavage and deprotection of Rink amide MBHA resin

After elongation and *N*-terminal capping was complete, the resin was washed with CH_2Cl_2 (5 × 2 mL × 2 min), Et_2O (5 × 2 mL × 2 min) and dried under vacuum for ca. 2 h. Peptides were simultaneously cleaved and side-chain deprotected using 'Reagent K' (TFA:EDT:Thioanisole:Phenol:H_2O 82:3:5:5:5; 3 × 2 mL × 2 h). The solution was precipitated in ice-cold Et_2O (25 mL) and placed in a centrifuge (3000 rpm × 10 min), the supernatant removed and the precipitate resuspended in ice-cold Et_2O and placed in a centrifuge again. This process was repeated 3-4 times and the precipitate was dried under a stream of nitrogen overnight, before being dissolved in H_2O and lyophilised.

Peptide Purification

In general, peptides were purified by automated RP column chromatography on a Biotage Isolera 1.3.3., using a RediSep®Rf gold reversed phase C18 column by Teledyne Isco on an increasing gradient of acetonitrile (5-50%) in water + 0.1% TFA (v/v) at a flow rate of 12 mL min^{-1}. Crude peptides were suspended in H_2O as concentrated as possible, fractions were checked by LCMS, concentrated *in vacuo* and lyophilised. Peptides were purified further by preparative UV- or MD- HPLC using a Jupiter Proteo preparative column (reversed phase) on an increasing gradient of acetonitrile in water + 0.1% formic acid (v/v) at a flow rate of 10 mL min^{-1}. Crude peptides were suspended in H_2O at an approximate concentration of 20 mg mL^{-1}. Purification runs injected a maximum of 0.9 mL of crude peptide solution and were allowed to run for 30 min, with acetonitrile increasing at a stated gradient. In regards to UV-HPLC, the eluent was scanned with a diode array at 220, 210 and 280 nm. For MD-HPLC, the mass directed chromatography software Masshunter by ChemStation (Agilent) was used to allow the collection of the desired peptide by mass, with the eluent split into an Agilent 6120 Quadropole LCMS which triggers collection of eluent at a programmed m/z. Fractions containing purified peptide were combined, concentrated *in vacuo* and lyophilised.

Peptide Characterization Data

Tabulated HRMS data of synthesised peptides are shown below. Peptide identity was confirmed by the inspection of multiple charge states and are quoted as the monoisotopic peak for the Expected (Exp[d]) and Observed (Obs[d]) masses.

Peptide	Sequence	$[M+4H]^{4+}$ Exp[d]	$[M+4H]^{4+}$ Obs[d]	Purity (%)
Wt-NOXA$_{75\text{-}93}$	AAQLRRIGDKVNLRQKLLN	562.50	562.91	90
NOXA$_{75\text{-}93}$L78F	AAQ**F**RRIGDKVNLRQKLLN	571.60	571.65	94
NOXA$_{75\text{-}93}$L78W	AAQ**W**RRIGDKVNLRQKLLN	581.19	581.09	94
NOXA$_{75\text{-}93}$L78Y	AAQ**Y**RRIGDKVNLRQKLLN	575.12	575.34	95
NOXA$_{75\text{-}93}$V85F	AAQLRRIGDK**F**NLRQKLLN	574.58	574.84	95

Peptide	Sequence	$[M+2H]^{2+}$ Exp[d]	$[M+2H]^{2+}$ Obs[d]	Purity (%)
Wt-$SIM_{2705-2717}$	DNEIEVIIVWEKK	828.45	828.40	100
$SIM_{2705-2717}$I2708D	DNE**D**EVIIVWEKK	829.42	829.43	96
$SIM_{2705-2717}$V2710I	DNEIE**I**IIVWEKK	835.46	835.52	97
$SIM_{2705-2717}$I2711E	DNEIEV**E**IVWEKK	836.43	836.44	98
$SIM_{2705-2717}$V2713E	DNEIEVII**E**WEKK	843.44	843.44	91

Protein Expression and Purification

h$MCL\text{-}1_{172-327}$ was expressed and purified as previously described from a pet28a His-SUMO Mcl-1 (172-327) construct with cleavage of the tag using Smt3 protease, Ulp1.[4]

h$SUMO\text{-}1_{18-97}$ was expressed and purified as previously described from a pet19b His-TEV-SUMO-1 (18-97) construct with cleavage of the tag using TEV protease.[4]

Fluorescence Anisotropy

Fluorescence anisotropy assays were performed in 384-well plates (Greiner Bio-one). Each experiment was run in triplicate and the fluorescence anisotropy measured using a Perkin Elmer EnVisionTM 2103 MultiLabel plate reader, with excitation at 480 nm (30 nm bandwidth), polarised dichroic mirror at 505 nm and emission at 535 nm (40 nm bandwidth, S and P polarised). All assays were performed in 20 mM Tris, 100 mM NaCl, 0.1 mM DTT, pH 7.5 unless otherwise stated and data analysed following previously published methods.

Fluorescence anisotropy data was processed as described previously.[2] Briefly. the data from both the P (perpendicular intensity) and S (parallel intensity) channels, resulting from this measurement and corrected by subtracting the corresponding control wells, were used to calculate the intensity and anisotropy for each well following Equations 1 and 2:

$$I=(2PG)+S \quad Equation\ 1$$

$$r=(S-PG) \quad Equation\ 2$$

For direct titration the average anisotropy (across three experimental replicates) and the standard deviation of these values were then calculated and fit to a logistic model using OriginPro 9.0 to determine the maximum and minimum anisotropies then the fraction bound calculated (Equation 3) and used to determine K_d (equation 4)

$$L_b=(r-r_{min})/\lambda(r_{max}-r)+r-r_{min} \quad Equation\ 3$$

$$y=\{(k+x+[FL])-\surd\{k+x+[FL]^2-4x[FL]\}\}/2 \quad Equation\ 4$$

r = anisotropy, I = total intensity, P = perpendicular intensity, S = parallel intensity, G = instrument factor which was set to 1 for all experiments, L_b = fraction ligand bound, λ = Ibound/Iunbound = 1, [FL] = concentration of fluorescent ligand, k = K_d , y = L_b* Flu-trimer and x = [added titrant].

For competition experiments, the anisotropies were determined as for the direct titration and then plotted against inhibitor concentration and fit (equation 5) to determine the IC_{50} and error values.

$y = r_{max} + (r_{min}-r_{max})/(1+(x/x_o)^p)$ $Equation\ 5$

Where x_o represents the IC_{50}

Direct binding assays:

Fluorescence anisotropy direct titration assays were performed with protein concentration diluted over 16-24 points using 2-fold dilutions. Followed by addition of tracer peptide was added to the wells. For control wells, the tracer peptide was replaced with an identical volume of assay buffer. Plates were read after 45 minutes.

Competition binding assays:

FA competition assays were performed in 384 well plates with the concentration of variant peptide competitor typically starting from 850 μM, diluted over 16 points using 2-fold dilutions with fixed protein and tracer concentrations. FITC-labelled peptide was added to each well to give a final concentration of 25 nM of FITC-Ahx-BIM_{75-85} or 50 nM of FITC-PEG-$SIM_{2705-2712}$. For control wells, the tracer peptide was replaced with an identical volume of assay buffer. The total volume in each well was 60 μL. Plates were read after 45 minutes of incubation at room temperature.

Isothermal Calorimetry

ITC experiments were carried out using a Microcal ITC200i instrument (Malvern) at 25°C in 20 mM Tris, 150 mM NaCl, pH 7.5 buffer. The protein of interest was dialysed against the buffer prior to experiment, and lyophilized peptides were dissolved in the same buffer. Protein was present in the cell and titrated with peptide solutions loaded into the syringe using 20 x 2 uL injections with 120 s spacing between the injections. Heats of peptide dilution were subtracted from each measurement raw data. Data was analysed using Microcal Origin 8 and fitted to a one-binding site model

Circular Dichroism

Spectra were recorded on a Chirascan circular dichroism spectropolarimeter (Applied Photophysics), at 20°C, using 1 mm cells and a scan speed of 5 nm/min. The spectra were averaged over 3 repeats with a buffer baseline subtracted. Peptide concentrations of approximately 0.1 mg/mL were used (although the exact concentration was used to allow determination of MRE). The solvent signal was subtracted to the raw circular dichroism data obtained for the peptides before conversion to the mean residue ellipticity (MRE).

$$[\theta]=\theta/10 \times c \times l$$

$$[\theta]_{MRE}=[\theta]/(R-1)$$

Where θ = circular dichroism at a given wavelength, *c* = molar concentration, l = path length in cm, R = number of residues in the peptide sequence.

Solubility Analyses

Peptide solubility was assessed using a qualitative test.

Proteolysis

All experiments were performed in triplicate in 50 mM Tris-HCl, pH 8.0, 150 mM NaCl, 10 mM $CaCl_2$ (the 'proteolysis buffer'), using 96-well flat-bottom assay plates. For α-chymotrypsin

($\varepsilon^{1\%}_{280}$ = 20.4 $M^{-1}cm^{-1}$), Proteinase K ($\varepsilon^{1\%}_{280}$ = 14.2 $M^{-1}cm^{-1}$), and the SIM variant peptides (ε_{280} = 5690 $M^{-1}cm^{-1}$), concentrations were determined based on the absorbance at 280 nm. For NOXA variants, concentrations were determined based on absorbance at 205 nm (ε_{205} = 58470 $M^{-1}cm^{-1}$, 67070 $M^{-1}cm^{-1}$, and 64550 $M^{-1}cm^{-1}$ for N-acetylated $NOXA_{75\text{-}93}$, L78F, and L78Y, respectively).[7]

An α-chymotrypsin (bovine pancreas) stock solution (1.5 mg/mL) was prepared by resuspension of the lyophilised protein (Sigma) in 10 mM HCl, and was then frozen in aliquots at -80 °C. For use in proteolysis assays, a single aliquot was thawed and diluted to 0.26 µg/mL in the proteolysis buffer, incubating at 25 °C for 2 hours before use. Proteolysis of select NOXA variant peptides (wt-NOXA, L78F, and L78Y) was performed with 45 µM peptide (10 µL of 1 mM stock solutions) in the presence of 0.25 µg/ml α-chymotrypsin (190 µL at 0.26 µg/ml) at 25 °C, in the proteolysis buffer. At specific time points, 20 µL of each proteolysis reaction was quenched in 80 µL 2% v/v trifluoroacetic acid (TFA).

A Proteinase K (*Tritirachium album*) stock solution (2.0 mg/mL) was prepared by resuspension of the lyophilised protein (Sigma) in the proteolysis buffer, and was then frozen in aliquots at -80 °C. For use in proteolysis assays, a single aliquot was thawed and diluted to 11.25 µg/mL in the proteolysis buffer. Proteolysis of SIM variant peptides (wt-SIM, I2708D, V2710I, I2711E, and V2713E) was performed with 40 µM peptide (280 µL of 43 µM stock solutions) in the presence of 0.75 µg/mL Proteinase K (20 µL of 11.25 µg/mL) at 25 °C, in the proteolysis buffer. At specific time points, 20 µL of each proteolysis reaction was quenched in 80 µL 2% v/v trifluoroacetic acid (TFA).

Quenched samples were analysed by LC-MS, using a Bruker maXis Impact QTOF mass spectrometer (electrospray ionization source), with a Dionex UltiMate 3000 liquid chromatography system (Thermo Scientific), equipped with a Waters Acquity Protein BEH C4 Column (300 Angstrom pore size, 1.7um particle size, 2.1mm x 50mm), running a gradient between water and acetonitrile, both supplemented with 0.1% formic acid. The area of the extracted ion chromatograms (EIC) for the full-length peptide of interest was used to determine the amount of proteolyzed material remaining (compared to the first time point – 3 min for NOXA variants, 2 min for SIM variants), monitoring the $[M+4H]^{4+}$ charge state for NOXA peptides and the $[M+2H]^{2+}$ charge state for SIM peptides.

Data were fitted to an exponential decay function:

$$y = (y_0 - y_F)e^{-\frac{x}{k}} + y_F$$

where y_0 and y_F are the y values at 0 min and at the plateau, respectively, and k is the normalized half time. For all data, y_0 was given a lower bounds limit of 0.

Peptide Analytical Data:

NOXA-B peptides

NOXA$_{75\text{-}93}$

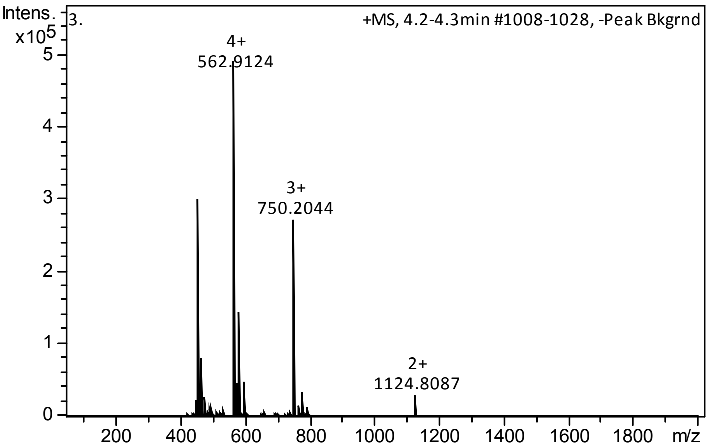

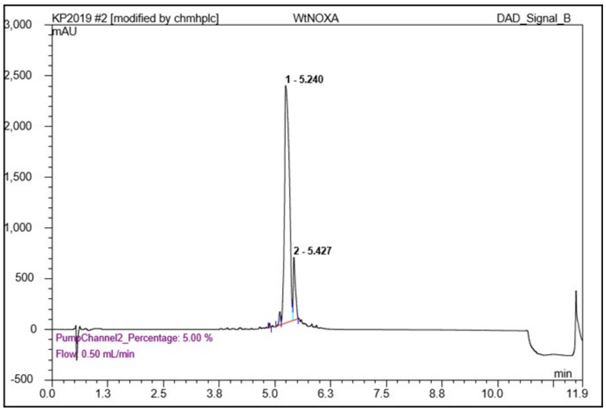

NOXA$_{75\text{-}93}$ L78F

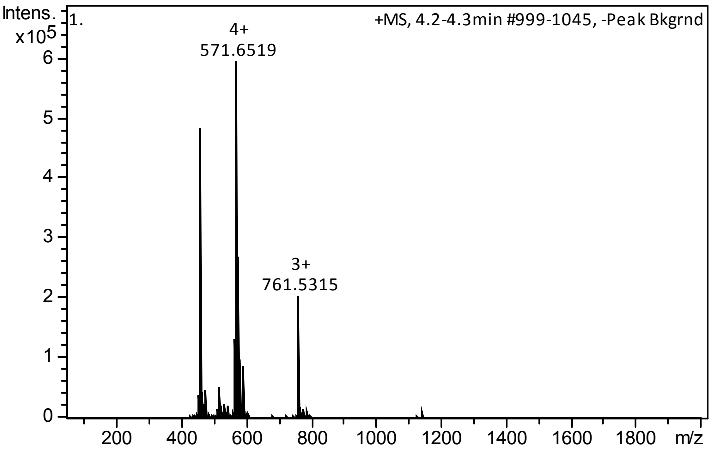

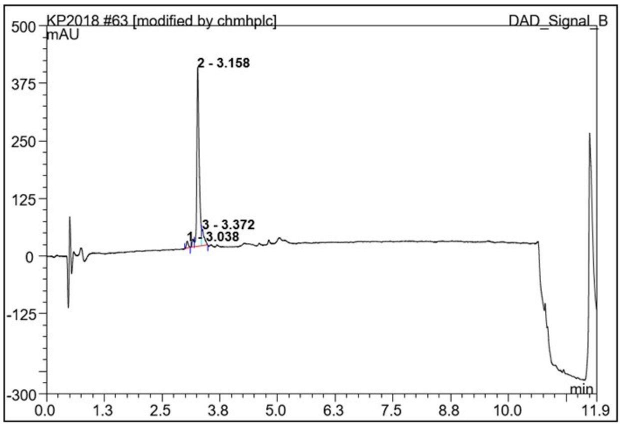

NOXA$_{75\text{-}93}$ L78W

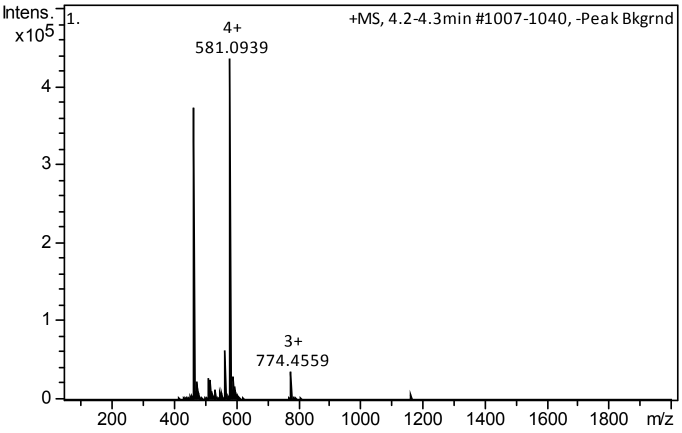

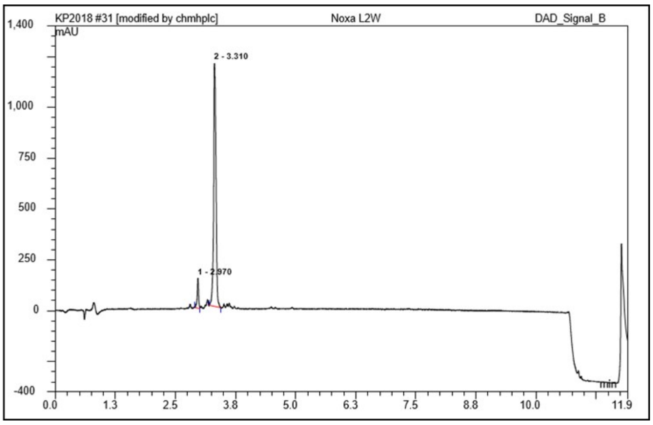

NOXA$_{75\text{-}93}$ L78Y

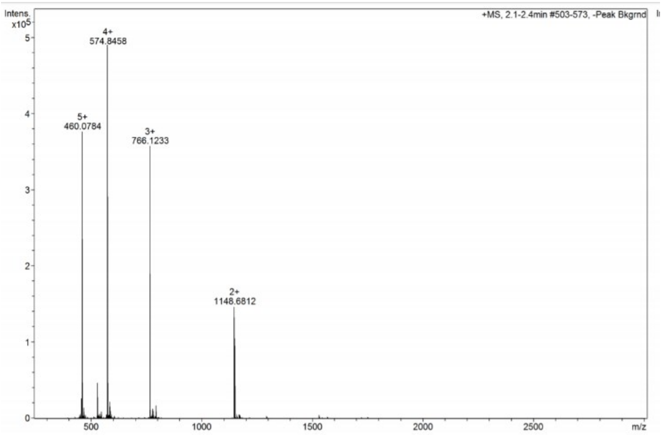

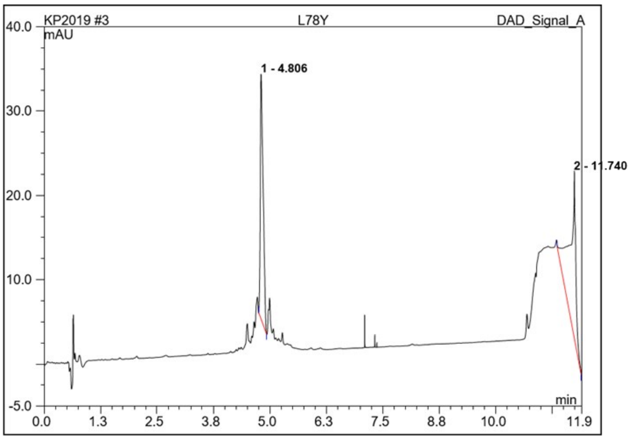

NOXA$_{75\text{-}93}$ V85F

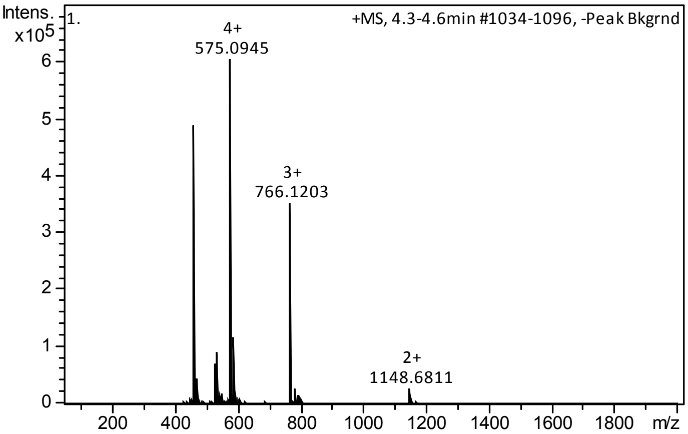

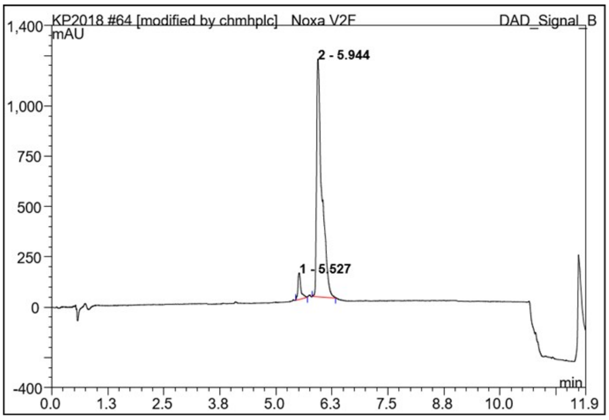

SIM peptides

SIM$_{2705\text{-}277}$

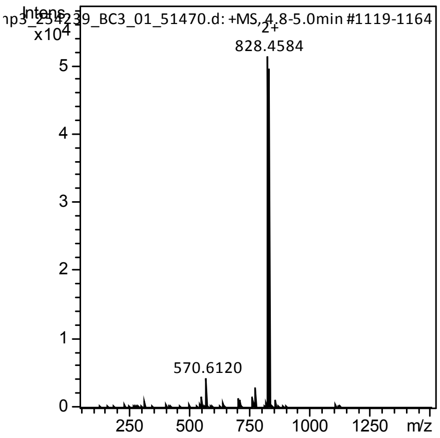

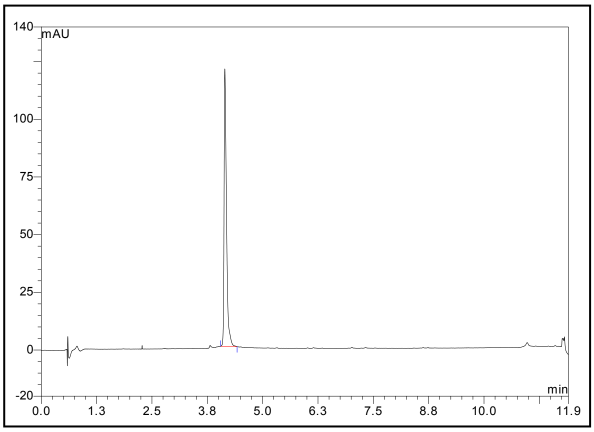

SIM$_{2705-277}$ I2708D

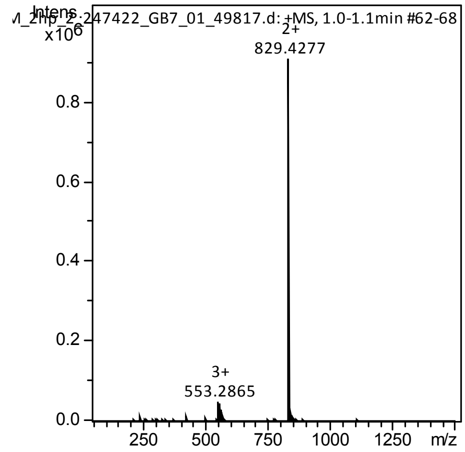

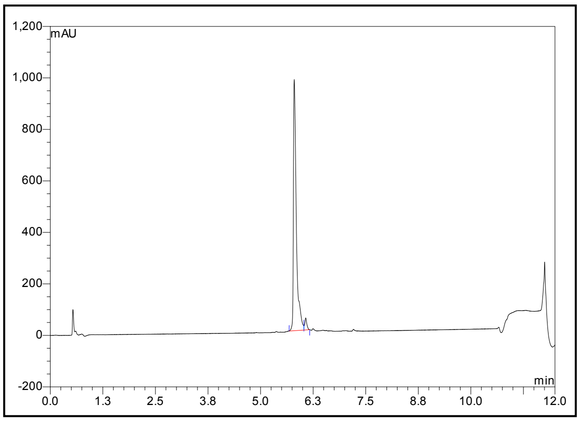

SIM$_{2705-277}$ V2710I

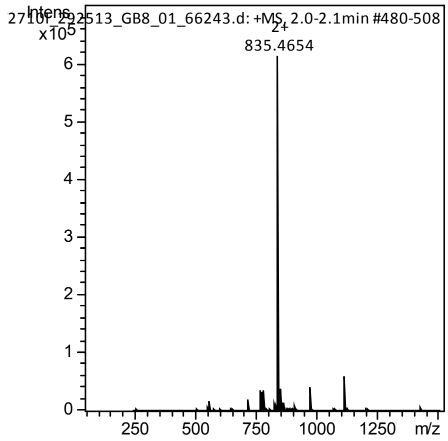

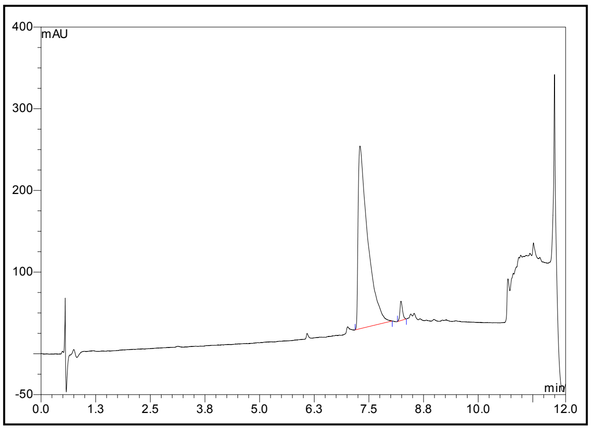

SIM$_{2705-277}$ I2711E

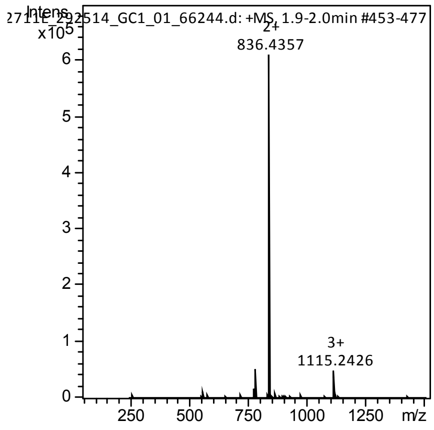

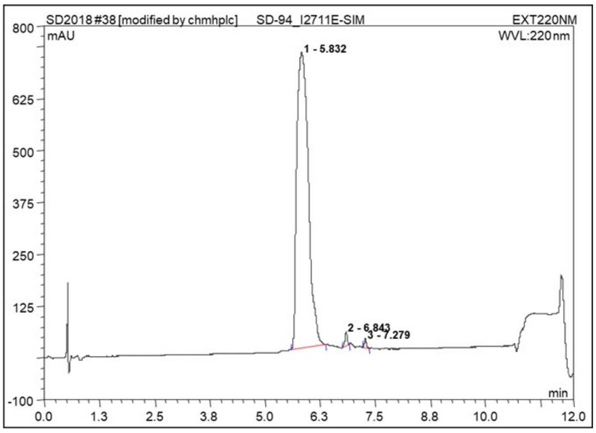

SIM$_{2705-277}$ V2713E

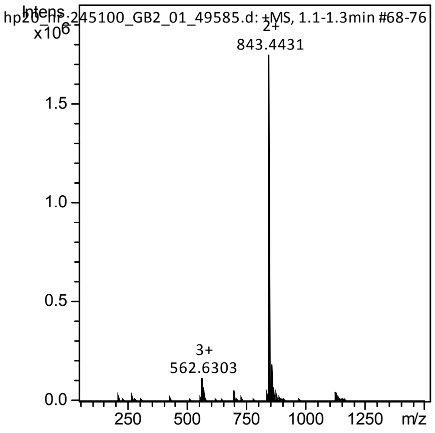

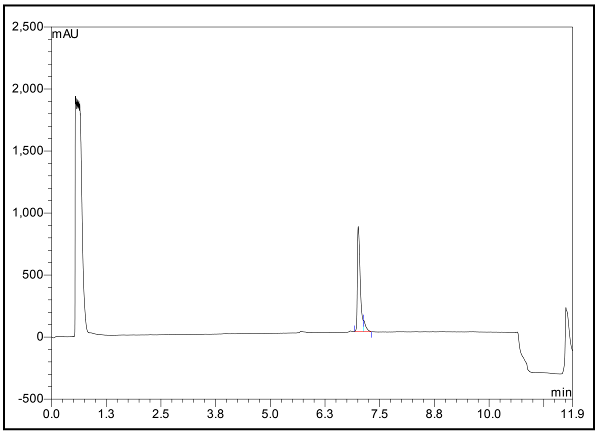

References

1. E. Gasteiger, C. Hoogland, A. Gattiker, S. Duvaud, M. R. Wilkins, R. D. Appel and A. Bairoch, in *The Proteomics Protocols Handbook*, ed. J. M. Walker, Humana Press 2005, pp. 571-607.
2. J. Kyte and R. F. Doolittle, *J. Mol. Biol.*, 1982, **157**, 105-132.
3. C. Nick Pace and J. Martin Scholtz, *Biophys. J.*, 1998, **75**, 422-427.
4. A. A. Ibarra, G. J. Bartlett, Z. Hegedüs, S. Dutt, F. Hobor, K. A. Horner, K. Hetherington, K. Spence, A. Nelson, T. A. Edwards, D. N. Woolfson, R. B. Sessions and A. J. Wilson, *ACS Chem. Biol.*, 2019, **14**, 2252-2263.
5. C. W. Wood, A. A. Ibarra, G. J. Bartlett, A. J. Wilson, D. N. Woolfson and R. B. Sessions, *Bioinformatics*, 2020, **36**, 2917-2919.
6. E. Kaiser, R. L. Colescott, C. D. Bossinger and P. I. Cook, *Anal. Biochem.*, 1970, **34**, 595-598.
7. N. J. Anthis and G. M. Clore, *Protein Sci.*, 2013, **22**, 851-858.
